# Missing Links: the Role of Primates in Understanding the Human Microbiome

**DOI:** 10.1128/mSystems.00165-19

**Published:** 2019-05-21

**Authors:** Katherine R. Amato

**Affiliations:** a Department of Anthropology, Northwestern University, Evanston, Illinois, USA

**Keywords:** gut microbiome, life history, primate

## Abstract

The gut microbiome can influence host energy balances and metabolic programming. While this information is valuable in a disease context, it also has important implications for understanding host energetics from an ecological and evolutionary perspective. Here I argue that gut microbial influences on host life history—the timing of events that make up an organism's life—are an overlooked but robust area of study given that variation in life history is linked directly to host energetic budgets and allocation patterns. Additionally, while cultural influences on life history complicate the exploration of these links in humans, nonhuman primates represent an alternative system in which more robust associations can be made. By integrating human and nonhuman primate microbiome research within the context of life history theory, we will be able to more effectively pinpoint microbial contributions to host phenotypes. This information will improve our understanding of host-microbe interactions in health and disease and will transform the fields of ecology and evolution more generally.

## Abstract (precis)

The gut microbiome can influence host energy balances and metabolic programming. While this information is valuable in a disease context, it also has important implications for understanding host energetics from an ecological and evolutionary perspective.

## PERSPECTIVE

A little over a decade ago, a group of transformative papers demonstrated that the gut microbiome plays a causative role in obesity (1, 2). One of the potential mechanisms identified was enrichment in microbial taxa that produce host-accessible energy in the form of short-chain fatty acids (SCFAs) more efficiently from host dietary carbohydrates. It was posited that individuals with these efficient microbiomes could obtain more energy from the same diet than individuals without them. Assuming hosts could not use this additional energy, it would be stored as fat and contribute to weight gain. The clinical implications of this research were immense, and the biomedical community jumped full throttle into the new field of microbially mediated metabolic disease.

**FIG 1:**
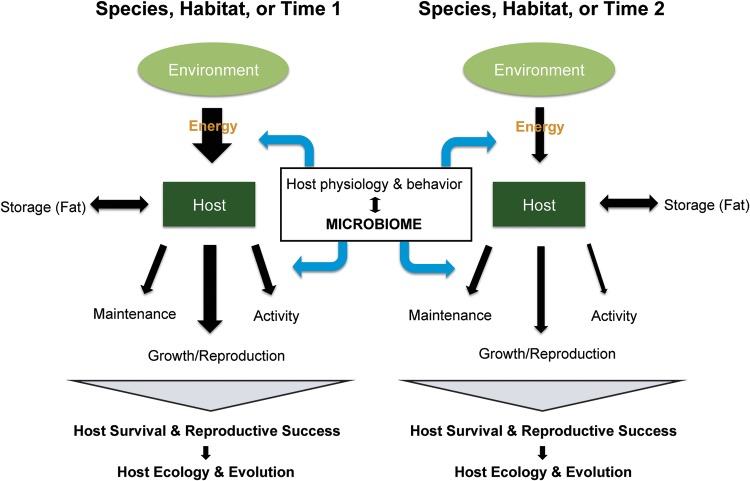
Life history theory dictates that hosts obtain a limited amount of energy from the environment (black arrows), which then must be allocated among the processes of maintenance, growth and reproduction, and activity. Energy can also be stored as fat to contribute to the overall energy budget at a later time. The overall energy budget and how it is allocated across processes can vary among host species and within host species across space and time, as depicted in these two cartoon examples. Traditionally, this variation has been described as a function of host physiological and behavioral traits. However, recent research implies that the microbiome can influence these dynamics as well through effects on host digestive efficiency and metabolic programming.

In the midst of this justified excitement, however, other potentially transformative applications of the results received less attention. In particular, many researchers—used to thinking in terms of metabolic disease and energy surplus—accepted the assumption that hosts would have no use for additional energy. And yet, large swaths of ecology and evolutionary biology are founded on the exact opposite assumption: organisms are energy limited, and their fitness depends on their ability to overcome energetic challenges. In particular, life history theory aims to understand the causes and consequences of variation in the timing and mode of life processes such as growth and reproduction (Fig. 1). A central tenet of life history theory is that organisms have access to a limited energy budget. Therefore, individuals, populations, and species must engage in a series of trade-offs to allocate this energy across processes, including growth, reproduction, and maintenance. Interactions of host environments and life history trade-offs shape patterns of reproductive success, ultimately determining host ecology and evolutionary trajectories.

In this context, the idea that intra- and interindividual variation in gut microbiomes could result in differential contributions to host energy budgets was new and exciting. Could gut microbial shifts be a mechanism by which hosts maintain growth or reproductive activity during seasonal periods of reduced food availability by increasing energy budgets? Could interindividual differences in this mechanism determine interindividual differences in fitness? What about the ability of different host species to survive in habitats with reduced nutritional resources? As an emerging ecologist studying wild primate nutrition and foraging behavior, these were the questions that most excited me. The idea that gut microbes could be contributing to host energy budgets was not new, particularly for the leaf-eating howler monkeys I was studying (3, 4), but it had not been formally integrated into models of primate bioenergetics. Reexamining primate bioenergetics and life history theory from a microbial perspective seemed like an obvious next step.

A decade later, the field of microbiome research has greatly expanded, and we now have a more nuanced understanding of the role of gut microbes in host energetics. Although certain gut microbes may contribute additional energy to their hosts, it also appears that gut microbes can directly program host metabolism through several potential mechanisms (5–8), determining how energy gets allocated in the host body (Fig. 1). While these interactions are mostly described in the metabolic disease literature, it is not a far leap to imagine similar microbial influences on normal host metabolic processes. Thus, beyond simply influencing whether a host will survive a period of food shortage, gut microbes likely play a central role in shaping host life history trade-offs and strategies.

Nevertheless, life history theory continues to be largely overlooked by the microbiome community in favor of more clinical approaches. Studies examining changes in the human gut microbiome during pregnancy and infancy as well as studies examining seasonality in the human gut microbiome have clear relevance for life history theory. In almost all cases, though, both the study design and data interpretation target health outcomes and do not directly address ecological or evolutionary implications.

Arguably, cultural influences on human life events such as reproduction mask biological processes and complicate the study of life history theory in human populations. Here, research with wild, nonhuman primates becomes indispensable. Data describing host-microbe dynamics and their influences on primate life history provide insight into mechanisms that were likely important in shaping the ecology and evolution of our human ancestors. While specific microbial taxa and functions of interest may differ, the general principles of interaction are likely to be conserved across host taxa and contexts. As a result, the primate literature represents a rich source of hypotheses and preliminary data that can subsequently be applied to the human context.

Several examples of potential microbial contributions to host life history, ecology, and evolution exist in the primate literature. A new area of “microbial reproductive ecology” in primate studies aims to understand how microbes might shape female reproductive success across species and environments (9). Additionally, a wide range of studies describes variation in the primate gut microbiome across time and space (e.g., see references [Bibr B10][Bibr B11][Bibr B15]). While, in some ways, these data sets are similar to those being generated for humans, study designs and data interpretation are almost exclusively framed in ecological and evolutionary contexts. For example, some of my work examines differences in the howler monkey gut microbiome across habitats with the ultimate goal of understanding how the gut microbiome and its interactions with host energetics influence host success in these habitats (14). Therefore, the potential role of gut microbes in influencing host life history strategies is much clearer in the primate literature than the human literature. However, the primate microbiome is grossly understudied compared to the human microbiome, and as a result, even here, links between the gut microbiome and host life history remain largely theoretical.

Beyond research focused solely on humans or primates, another rich opportunity for understanding microbial influences on host life history is comparative work. Compared to other primates, humans have large brains and exhibit a unique suite of life history traits. For example, humans have long life spans with extended periods of juvenile dependency. As a result, the high energetic demands of growing and maintaining our brains must be fulfilled across seasons and years, increasing the likelihood of nutritional stress due to periods of food shortage. At the same time, interbirth intervals are shorter than expected, which may further increase female energetic demands during the nutritionally risky periods of pregnancy and lactation. Therefore, microbial contributions to human energetic budgets and metabolic programming might be critical for facilitating human survival and reproduction, particularly in variable environments. In this context, identifying human microbial traits that are unique among primates and linking them to unique human physiological traits could be transformative for understanding the evolution of human physiology and life history. Nevertheless, systematic, comparative studies of human and nonhuman primate microbiomes and their links to host life history are essentially nonexistent in the literature.

Understanding microbial contributions to host life history has the potential to greatly advance the fields of animal ecology and evolution. We know that the gut microbiome is strongly influenced by the host environment. If microbes associated with a given environment contribute to the emergence of specific host phenotypes, discussions of host phenotypic plasticity and local adaptation must begin to consider host-gut microbe interactions. Given that human evolution was characterized by exposure to a diverse range of novel environments, these processes may provide important insight into the mechanisms behind human evolutionary success. There may also be implications for understanding microbiome plasticity and impacts on host physiology in modern human populations.

Additionally, an evolutionary perspective on host-microbe interactions can inform clinical work. Knowledge of our deep microbial history will help us consider how changes in human cultures, lifestyles, and environments over multiple timescales have interrupted evolved host-gut microbe relationships. This information can elucidate factors that predispose humans to disease via impacts on the microbiome in ways that traditional biomedical approaches cannot. Specifically, as more data describing host environment, physiology, and the microbiome are integrated from diverse human and nonhuman primate populations, it will allow us to distinguish key microbial processes that are (i) shared among all primates, (ii) unique to humans, and (iii) vary across human populations. This information will allow us to more effectively design therapies and interventions that are intermediate between “one size fits all” and “personalized medicine.” Instead, we can target specific groups of people based on a deeper understanding of their ecology and evolutionary history as well as recent shifts in environmental, cultural, and economic contexts.

In conclusion, incorporating a life history perspective into gut microbiome research has the potential to lead to transformative advances in ecology and evolution as well as medicine. By using a systems perspective to integrate microbial functions into existing models and theories, the microbiome community can address long-standing gaps in multiple fields while simultaneously contributing to mechanistic knowledge of host-microbe interactions. While current studies in human research provide some opportunity for these approaches, the explicit integration of life history theory into new study designs as well as the complementary use of wild, nonhuman primates will allow us to more directly address questions with implications for host ecology and evolution. The resulting data are also likely to feed back to influence biomedical research, similar to that which originally inspired this line of inquiry. In this way, using gut microbiome research to inform life history theory exemplifies the robust interdisciplinary nature of the field and emphasizes the importance of integrating multiple data sets and approaches. Broader adoption of these cross-cutting methods and approaches will promote the continued rapid advance of host-microbe science.
